# Extracellular Vesicles from Airway Secretions: New Insights in Lung Diseases

**DOI:** 10.3390/ijms22020583

**Published:** 2021-01-08

**Authors:** Laura Pastor, Elisabeth Vera, Jose M. Marin, David Sanz-Rubio

**Affiliations:** 1 Translational Research Unit, Instituto de Investigación Sanitaria de Aragón (IISAragón), Hospital Universitario Miguel Servet, 50009 Zaragoza, Spain; lpastorbernad@gmail.com (L.P.); eliverasolsona@hotmail.com (E.V.); jmmarint@unizar.es (J.M.M.); 2 Respiratory Service, Hospital Universitario Miguel Servet, University of Zaragoza, 50009 Zaragoza, Spain; 3 Centro de Investigación Biomédica en Red de Enfermedades Respiratorias (CIBERes), 28029 Madrid, Spain

**Keywords:** extracellular vesicle, COPD, asthma, fibrosis, induced sputum, nasal lavage, oral lavage, bronchoalveolar lavage, exosome

## Abstract

Lung diseases (LD) are one of the most common causes of death worldwide. Although it is known that chronic airway inflammation and excessive tissue repair are processes associated with LD such as asthma, chronic obstructive pulmonary disease (COPD) or idiopathic pulmonary fibrosis (IPF), their specific pathways remain unclear. Extracellular vesicles (EVs) are heterogeneous nanoscale membrane vesicles with an important role in cell-to-cell communication. EVs are present in general biofluids as plasma or urine but also in secretions of the airway as bronchoalveolar lavage fluid (BALF), induced sputum (IS), nasal lavage (NL) or pharyngeal lavage. Alterations of airway EV cargo could be crucial for understanding LD. Airway EVs have shown a role in the pathogenesis of some LD such as eosinophil increase in asthma, the promotion of lung cancer in vitro models in COPD and as biomarkers to distinguishing IPF in patients with diffuse lung diseases. In addition, they also have a promising future as therapeutics for LD. In this review, we focus on the importance of airway secretions in LD, the pivotal role of EVs from those secretions on their pathophysiology and their potential for biomarker discovery.

## 1. Introduction

Lung diseases (LD) are among the most common causes of death and disability worldwide [1]. According to the last updates of the Forum of International Respiratory Societies (FIRS), the high incidence of those pathologies is mainly due to global exposure to polluted air, particles, chemicals and infectious microorganisms [1]. Repeated exposures to these agents induce cell damage and trigger immune responses in the airway. Airway epithelium, which is the first line of defense against environmental agents, is damaged by this contact. Interactions and homeostasis between airway epithelium and mesenchymal, endothelial and immune cells in the airway and lungs are also modified [2,3]. Chronic airway inflammation and excessive tissue repair processes result in the development of LD such as asthma, chronic obstructive pulmonary disease (COPD), idiopathic pulmonary fibrosis (IPF) or lung cancer, as well as in systemic inflammation and associated co-morbidities [3,4,5].

Extracellular vesicles (EVs) are heterogeneous nanoscale membrane vesicles, including exosomes, microvesicles and apoptotic bodies, that are secreted by most cell types and they have been mainly described in plasma and serum [6,7,8,9] or urine. However, the presence of EVs has been also reported in secretions from airway such as saliva [10], induced sputum (IS) [11] or bronchoalveolar lavage fluid (BALF) [12]. EVs have emerged as a novel and crucial mechanism of intercellular communication [13,14] with an important role in inflammatory pathologies, including LD. Airway injury modifies EV cargo in lung epithelium derived-EVs promoting an immune response via macrophage activation [15,16]. Therefore, EVs could contribute to clarify the molecular mechanisms underlying LD, but also they could provide new biomarkers among their cargo, as well as new EV-based therapeutic approaches [17,18].

In this review, we aim to summarize current knowledge about EVs related to LD. We will focus on the main LD such as COPD, asthma and IPF, and on the potential of airway secretions as source of new biomarkers. Finally, we suggest the potential function of EVs from these secretions in the pathophysiology of LD and their promising future as origin of biomarkers and for LD.

## 2. Lung Diseases

### 2.1. Asthma

Asthma is one of the most prevalent chronic disease in developed countries in adults and children globally [1], affecting more than 339 million people [19] and 14% of children over the world [1]. Asthma is defined by the international Global Initiative for Asthma (GINA) as a heterogeneous disease related to chronic airway inflammation and characterized by intermittent episodes of wheeze, cough, dyspnea and chest tightness, as well as reversible airway limitation [19]. This disease shows different phenotypes and endotypes, depending on its mechanisms and responses to therapy [20].

**Figure 1:**
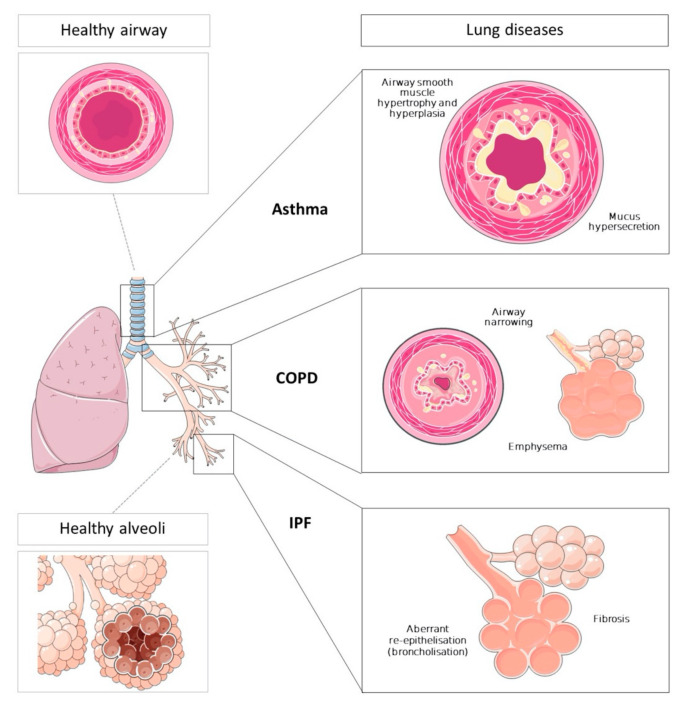
Overview of the main processes and events that occur in different regions of lung and airway in asthma, chronic obstructive pulmonary disease (COPD) and interstitial pulmonary fibrosis (IPF) patients, compared to healthy condition. Asthma involves mainly the upper airway promoting airway remodeling, which includes airway smooth muscle hypertrophy and hyperplasia, and goblet cell hyperplasia and consequent mucus hypersecretion. These processes cause reversible airway narrowing. COPD affects lower airways and lung parenchyma causing irreversible airway narrowing and alveolar disruption (emphysema) in the lungs. IPF affects only the lungs through excessive fibrosis processes with subsequent aberrant alveolar re-epithelisation (broncholisation). (Components of this figure have been obtained and modified from Servier Medical Art, https://smart.servier.com, with permission under the Creative Commons Attribution 3.0 Unported License.).

Inflammation in asthma is extended from upper to peripheral airway revealing different molecular patterns. Most of the patients suffer allergic asthma characterized by Th2 immune response against environmental stimuli [21]. After antigen presentation, which is driven by dendritic cells [22], T-CD4^+^ lymphocytes secrete Th2-cytokines (IL-4, IL-13 and IL-5). IL-4 and IL-13 stimulate IgE production in B-cells [23], while IL-5 promotes eosinophil maturation in bone marrow and further eosinophil recruitment in the airway [21,24]. Non-allergic T2-eosinophilic response is pushed by alarmins and innate lymphoid cells 2, which are producers of Th2-cytokines, so they trigger eosinophilic inflammation in airway [25]. Finally, non-eosinophilic phenotype is normally related to more severe status and it is generally characterized by neutrophilic airway inflammation [25,26]. Although it is known that non-eosinophilic asthma pathophysiology is driven by Th17/Th1 cells [25] and cytokines as IL-17 and IL-22 [26], its molecular mechanisms are poorly understood. Airway epithelial injury produced by this inflammatory situation triggers asthma-characteristic airway remodeling, including the increase of airway smooth muscle bulk and mucus hypersecretion. Altogether, as shown in Figure 1, these phenomena produce airway narrowing and airway hyperresponsiveness to some environmental agents, which is also a characteristic feature of asthma [27,28].

### 2.2. Chronic Obstructive Pulmonary Disease

COPD has a noteworthy impact on global health, affecting more than 200 million people worldwide [1], leading mortality (3rd cause of death) and morbidity in the world [1,29]. COPD is a LD characterized by persistent airflow limitation and respiratory symptoms due to airway and/or alveolar damage. It is usually caused by significant exposure to irritants, especially cigarette smoke, although sometimes is influenced by host factors as abnormal lung development or α_1_-antitripsin genetic deficiency [30]. COPD is associated to systemic inflammation, which is a risk factor to develop co-morbidities such as atherosclerosis and other cardiovascular diseases, diabetes, metabolic diseases or cancer [31,32].

In contrast to asthma, inflammation in COPD is limited to peripheral airways and lung parenchyma, resulting in fibrosis, tissue destruction, small airway obstruction and emphysema [33]. These mechanisms are irreversible and produce progressive decline in lung function [5]. Different cells are involved in COPD inflammation processes: adaptative immune cells, innate immune cells (mainly neutrophils and macrophages) and structural cells. In response to cigarette smoke or other noxious particles, airway epithelial cells and macrophages are activated to produce several inflammatory mediators [34]. The transcription factor NF-κβ, activated in alveolar COPD macrophages [35], up-regulates the expression of inflammatory molecules [36]. Neutrophils from peripheral airway release CXCL8, promoting autocrine neutrophil recruitment and several proteinases, which contribute to loss of elasticity and peripheral airway collapse. This processes, together with profibrotic effect of TGF- β released from epithelial cells and macrophages, result in alveolar destruction, fibrosis and irreversible airway narrowing [36] (Figure 1). Lungs of COPD patients also suffer an accelerated ageing produced by an abnormal accumulation of senescent structural cells [37]. Finally, eosinophils have been reported to be increased in airway of some COPD patients, suggesting the existence of a different COPD phenotype whose molecular mechanisms are poorly understood [38].

### 2.3. Idiopathic Pulmonary Fibrosis

IPF is increasing over the time worldwide with an estimated incidence of 2.8–18 cases/100,000 people per year in Europe and North America. Lower incidence is defined in Asia and South America, which may be partially due to under-diagnosis [39]. In addition, IPF is more frequent in men [40]. IPF is a chronic, progressive fibrosing interstitial pneumonia of unknown etiology that usually affects to older adults. Disease progression varies between patients and is unpredictable, but IPF leads to severe pathological processes which end in premature death [41].

Molecular mechanisms of IPF are not fully understood although chronic dysregulation in alveolar epithelial cells homeostasis has been described (Figure 1). IPF biopsies show loss of type 1 alveolar epithelial cells (AEC1s) and reduced abnormal renewal capacity in type 2 (AEC2s) [42,43]. Fibroblast aggrupation are also observed between abnormal AECs [44,45]. In response to these microinjuries, remaining AECs are activated to release fibrogenic growth factors and cytokines, resulting in the recruitment of myofibroblast from different sources [45,46,47]. At this location, myofibroblasts increase secretion of abnormal extracellular matrix components, whose accumulation and deposition leads to fibrosis and gas exchange impairment. It also enhances myofibroblast activation, establishing a positive feedback process [48]. In addition, peripheral airway basal cells act as stem cells to repair damaged alveolar airway with new epithelial cells, known as broncholisation [49,50,51].

## 3. Airway Secretions

Sampling the upper and lower airways is a remarkably helpful tool in clinical practice. Different airway secretions are used and has been lately investigated as a promising source of biomarkers, including bronchoalveolar lavage fluid (BALF), induced sputum (IS), nasal lavage fluid (NL) or pharyngeal lavage fluid (PHAL).

### 3.1. Bronchoalveolar Lavage Fluid

BALF is an established procedure for washing the bronchial tree with saline which allows sampling peripheral airways [52]. It is an invasive method, as it is performed together with a flexible fiberoptic bronchoscopy. BALF contains the cells in the alveolar space which have been in contact with the instilled fluid (alveolar macrophages, CD4^+^ and CD8^+^ lymphocytes, neutrophils, eosinophils and mast cells [53]), airway epithelial cells (contaminating BALF if exceed 5% of total cell count [53]), and different soluble components from the endogenous fluid in the alveoli, named the epithelial lining fluid (ELF) [54]. These components proceed mainly from local cell secretory production [55,56,57], but also from the vascular space in contact with the alveolar space [54]. In clinical practice, BALF is used in diagnosis of respiratory infections [58] and as a part of the diagnosis or in characterization of non-infective LD, both in adults and children [59]. However, its invasive nature has limited its use as a research tool.

### 3.2. Induced Sputum

**Figure 2:**
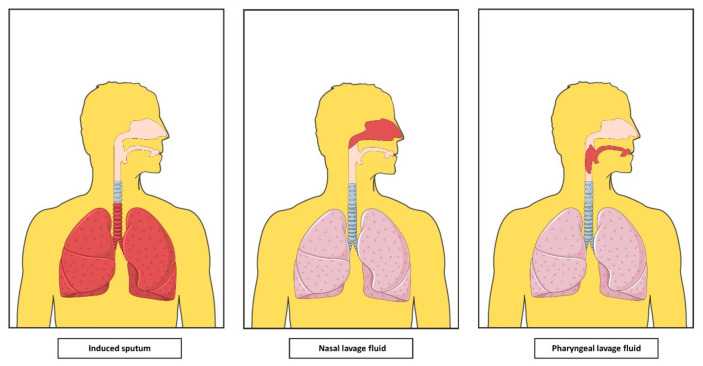
Anatomical origin of minimally invasive and non-invasive airway secretions. Induced sputum comes from lower airway while nasal lavage and pharyngeal lavage have their origin in different parts of the upper airway. (Components of this figure have been obtained and modified from Servier Medical Art, https://smart.servier.com, with permission under the Creative Commons Attribution 3.0 Unported License.).

Sputum induction is a relatively non-invasive method to collect a sample of cells and secretions from lower airways as shown in Figure 2 [60]. Sputum is induced by inhalation of hypertonic or isotonic sterile saline solution and obtained by further direct expectoration [61]. IS is a safer and less invasive method to sample ELF than BALF [62,63]. However, IS contains also saliva and squamous cells from upper airways, which are considered contaminant components and represent different proportions of IS [64,65].

Most of IS research has been dedicated to asthma, although multitude of studies have investigated the utility of IS in other LD, especially COPD and chronic cough [66]. Differential cell count in IS is used in clinical practice to assess asthma phenotypes [67], although reference parameters vary among different studies and populations [68,69,70]. In both asthma [71] and COPD [72], cultures from IS are performed to identify the presence of pathogens and manage antibiotic therapy. Extended biomarkers research has been done in IS. For example, assessment of inflammatory pattern of exacerbations in asthma and COPD by IS cytology allows therapy adjustment and prediction of new exacerbation episodes [73]. IS can work as a minimally invasive BALF surrogate in assessment of low airway status in LD with low airway involvement, although further research is needed in order to optimize protocols and standardize reference parameters. In this way, IS has been proposed and studied as a non-invasive surrogate of BALF in interstitial LD diagnosis [74].

### 3.3. Nasal Lavage Fluid

NL is a non-invasive procedure to obtain cytology from nasal cavities [75] (Figure 2). Different nasal lavage techniques have been described [76,77] consisting on instill sterile saline solution. NL is a mixture of saline and nasal secretions, containing epithelial cells, neutrophils, eosinophils, lymphocytes and their secreted mediators [78].

NL has been applied to assess the inflammatory status of a specific part of upper airways, the nasal cavities. Most NL studies have been focused in rhinitis, as it is a disease characterized by nasal mucosa inflammation and nasal hypersecretion [75,78,79]. Recent studies have shown that NL cytology is a best substitute for IS cytology than blood cell count in identification of inflammatory phenotypes of asthma [80,81]. In addition, despite the weaker relationship between nasal mucosa inflammation and COPD, another work revealed that IL-8 concentration in NL have positive correlation with disease severity and cigarette pack-years in COPD patients [82]. NL has a great potential as a non-invasive substitute of IS in those patients or situations where perform IS could be risked.

### 3.4. Pharyngeal Lavage Fluid

PHAL is a recently developed non-invasive technique to collect cells from the oropharyngeal cavity to assess mucosal inflammation of the upper airway [83]. Figure 2 shows that PHAL contains cells from the oropharyngeal mucosa (a major proportion of squamous epithelial cells, followed by a high percentage of neutrophils and other inflammatory cells [83]) and soluble factors and proteins [84].

Higher PHAL lymphocytes number have been found in the patients with obstructive sleep apnea (OSA) [85,86]. Our group reported also that T-CD4^+^cells number in PHAL correlated with more severe OSA status, as well as PHAL concentration of IL-6 and IL-8 [84]. Thus, PHAL is emerging as a new non-invasive tool to assess upper airway inflammation, which could be particularly helpful in some LD with concomitant upper airway inflammation such as asthma.

## 4. Extracellular Vesicles

EVs are a heterogeneous population of small membranous structures released into the extracellular environment by most cells and participate in intercellular communication [87]. EVs have been classified according to different criteria as their size or biogenesis, differentiating between exosomes, microvesicles (MVs), and apoptotic bodies (ABs) [88,89,90]. Exosomes (30–150 nm) result from a complex biogenesis which involves the endosomal membrane invagination, generating intraluminal vesicles (ILVs) inside the late endosome (MVB), and the fusion between the MVB membrane and the plasma membrane releases exosomes [91,92]. MVs (100 nm to 1000 nm), also named ectosomes or microparticles, are generated directly by outward budding of the plasma membrane [93,94]. ABs (1 to 5 µm) are released undergoing apoptosis, via plasma membrane bubbling or fragmentation due to cell disassembly [95,96].

EVs contain biomolecules from their origin cell, including proteins, lipids, metabolites and different nucleic acids (DNA, mRNA, miRNA and other noncoding RNA) [89,97,98]. Their molecular cargo can be uploaded into the vesicles selectively, mostly in exosomes. This process depends on cell status and type [99]. EV release has been reported in platelets [93], tumor cells [100], dendritic cells [101], T [102] and B [103] cells, eosinophils [104], epithelial cells [15,16], endothelial cells [105] or mesenchymal stem cells (MSCs) [106]. Their accessibility in body fluids (plasma [6,8], serum [9], urine [9], saliva [10], BALF [12] or IS [11]) and the specificity of their cargo associated with pathophysiological conditions [107,108] confer them great potential as biomarkers.

EVs have been broadly studied because of their association with pathologies, including inflammatory diseases [109,110] cardiovascular diseases [111,112], metabolic diseases [113,114], cancer [115,116] or LD [107,117,118]. They have been linked to pro-inflammatory processes, participating in antigen presentation [101,103] or macrophage activation [15], as well as to immunomodulatory processes [106]. Additionally, EVs could have a therapeutic approach as vehicles for drug delivery, given their intrinsic biocompatibility and specific target activity [119]. Promising examples of it are novel exosome-based modality of cancer therapy [120] or nanoparticle-guided asthma therapy [121].

In the last decade, the research on EVs has increased. However, the optimal isolation method remains unclear and most times depends on the downstream analyses [13,122,123,124]. Their characterization requires follow several points that include protein markers, size or morphology. Those are described in the MISEV2018 [87] updating the MISEV2014 [125], showing that the EV field is continuously in development and requires further stu-dies.

## 5. EVs in Lung Pathophysiology

Most lung cells release EVs under physiological and pathological conditions. Bronchial epithelial cells (BECs) and alveolar macrophages are the major sources of pulmonary EVs [126], but also vascular endothelial cells, fibroblasts, MSCs and dendritic cells. All together are involved in lung homeostasis, as well as in LD development.

Transfer of EVs cargo between airway epithelial cells can alter the secretions of their target cells, including mucins [127]. Membrane associated mucins are present in tracheobronchial epithelial cells exosomes surface, suggesting their contribution to innate mucosal defense of airway [128]. Similarly, alveolar macrophages release EVs that contain the suppressor of cytokines signaling (SOCS) 1 and 3. These EVs have immunomodulatory effects in the alveolar epithelial cells and their secretion is diminished by cigarette smoking [129]. Table 1 shows some of the proteins, lipids and miRNAs contained in EVs that have been related with asthma, COPD and IPF.

### 5.1. EVs in Asthma

**Figure 3:**
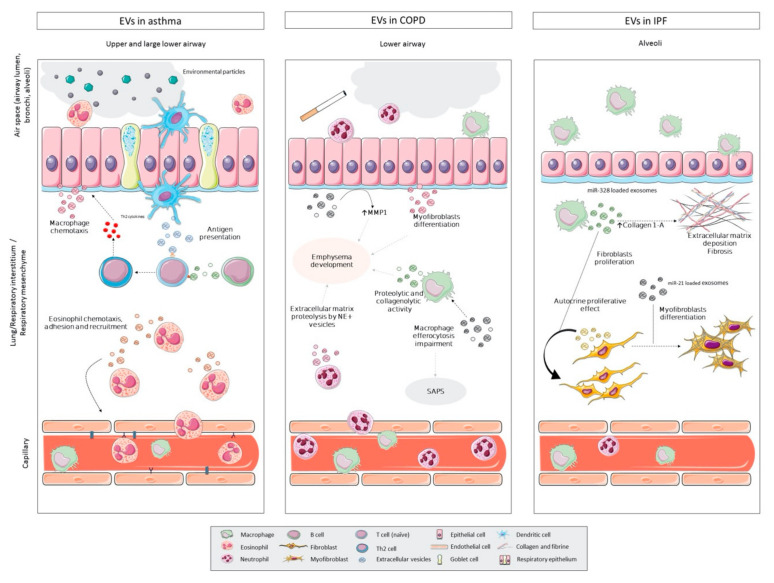
Summary of some of the main reported roles of extracellular vesicles (EVs) in the pathophysiological mechanisms of asthma, chronic obstructive pulmonary disease (COPD) and interstitial pulmonary fibrosis (IPF). The figure shows the cellular origin of the EVs as well as their cellular destiny or their main action. In the upper and large lower airway of asthma patients, EVs contribute to antigen presentation, EVs from endothelial cells participate in macrophage chemotaxis while eosinophil EVs could act in their own chemotaxis, adhesion and recruitment. In COPD, EVs could promote emphysema development increasing MMP1 secretion in lung epithelial cells, through myofibroblast differentiation or enhancing the proteolysis of the extracellular matrix. In the alveoli of IPF patients, EVs from macrophages could increase the collagen 1-A expression in fibroblast, increasing the extracellular matrix deposition, as well as promote fibroblast proliferation. (Components of this figure have been obtained and modified from Servier Medical Art, https://smart.servier.com, with permission under the Creative Commons Attribution 3.0 Unported License.).

EVs are involved in several processes of asthma immunity and inflammation. One of the most important is antigen presentation ability of EVs released from dendritic cells [101] and B cells [103,130]. Figure 3 shows that MHC-II and co-stimulatory molecules in the surfaces of these EVs allow interaction with T cells and subsequent differentiation of T cells, proliferation of T-CD4^+^ Th2 cells and secretion of Th2 cytokines [101,130].

Asthma-like inflammation driven by IL-13 enhances exosomes secretion in BECs inducing proliferation and chemotaxis of undifferentiated macrophages in the lungs [126]. Bronchoconstriction mechanical stress also stimulates exosomes secretion from BECs, producing typical subepithelial angiogenesis [146]. Furthermore, mechanical signals regulate miRNA cargo of EVs released from BECs [147]. In contrast, EV immunomodulatory effects has been reported to be diminished in asthma, such as EV-mediated transport of SOCS3 from alveolar macrophages to BECs [148].

Eosinophil exosomes of asthmatic patients differ their capacities from non-asthmatic subjects. They promote eosinophil chemotaxis increasing ROS and nitric oxide (NO) levels, and adhesion, via ICAM-1 and integrin-α_2_ upregulation [149] (Figure 3). These exosomes also promote apoptosis and reduce wound healing capacity in BECs via *TNF* or *CCL26* upregulation [150]. In addition, their angiogenic effect on bronchial smooth muscle cells is mediated by *VEGFA* [150]. Similarly, exosomes from neutrophils enhance neutrophils chemotaxis through their leukotriene B4 cargo [136] and adhesion by ICAM-1 upregulation in lung endothelial cells [151].

Few studies describe the effect of asthma pathophysiology on EV-miRNAs [152]. One of them described the increase of miR-223 and miR-142a on EVs from BALF of allergen-treated mice [138] while other, performed on asthmatic patients, described 24 miRNAs differentially expressed including members of let-7 and miR-200 families and miR-21 [139]. MiR-21 targets the pro-inflammatory Th1 cytokine, IL-12, and downregulate Th2 response, which is characteristic of asthma inflammation [153]. Accordingly, miR-21 is increased in serum of asthmatic patients and inversely correlated with FEV_1_ [154]. Lastly, a set of EV-miRNAs from BALF and miRNAs from lung tissue were altered in allergen-exposed mice and were inversely correlated between both origins [155].

### 5.2. EVs in COPD

EVs may also play a main role in COPD pathophysiology. One of the factors associated with COPD, cigarette smoke (CS), modifies EV cargo from different lung cells. CS induces significant release of CCN1-enriched exosomes from BECs, as well as the cleavage of matrix-associated CCN family protein into a shorter isoform [156], which promotes MMP1 secretion in lung epithelial cells contributing to emphysema development [156,157,158] (Figure 3). Similarly, macrophages exposed to CS release MVs with proteolytic and collagenolytic activity in their surface as shown in Figure 3 [159]. In addition, the increased macrophage-MVs release upregulates several pro-inflammatory and chemoattractant mediators, as ICAM-1, IL-8 and MCP-1, in alveolar epithelial cells [160]. Other studies have focused on EV non-coding RNAs in smokers and COPD patients. MiR-210 is overexpressed in both BEC-EVs and lung tissue samples after CS exposure. miR-210 loaded-EVs from COPD patients showed the induction of lung fibroblasts differentiation into myofibroblasts by silencing the autophagy-related factor *ATG7* [140]. Different profiles of long non-coding RNAs (lncRNAs) were found in circulating exosomes from non-smokers and smokers of different smoking products. EV-lncRNAs alterations found in smokers could lead to pulmonary fibrosis and mitophagy, which are hallmark processes in COPD and IPF. In addition, the targets of some of the differential lncRNAs are involved in pathological processes of COPD, IPF and asthma [161].

Overexpression of exosomal miR-21 from BECs also promotes myofibroblast differentiation targeting hypoxia-inducible factor 1α (HIF-1α) [141]. However, decreased miR-21 was observed in BEC exosomes exposed to CS. This compensatory mechanism in response to CS reduced M2 macrophage polarization and, consequently, epithelial-mesenchymal transition involved in airway remodeling [162]. Finally, CS induce on endothelial cells the production of MVs enriched in let-7d, miR-191, miR-126 and miR-125a, whose major effect in recipient macrophages was the impairment of efferocytosis [163].

Surface proteins identified in circulating endothelial MVs suggest that endothelial MVs have an important regulatory role in the coagulation and inflammation processes in COPD [164]. It has been also reported that EV-mediated transport of α_1_-antitrypsin from endothelial cells to alveolar epithelial cells is impaired by CS exposure [131]. In addition, exosomes from activated neutrophils contain neutrophil elastase (NE) in their surfaces, protecting NE from α_1_-antitrypsin proteolysis, and enabling the degradation of the extracellular matrix via NE^+^ exosomes [132] (Figure 3). These results suggest that endothelial and neutrophil derived-EVs contribute to emphysema development in COPD.

### 5.3. EVs in IPF

EVs implication in IPF remains unclear. Up to now, mostly EV studies related to mechanisms involved in fibrosis development used in vitro or murine models and focused their miRNA cargo. MiR-21 is upregulated in EVs from serum of a lung fibrosis mice model and from human IPF patients and it is correlated with IPF progression and mortality [143]. Another study showed that miR-21 upregulation is promoted via TGF-β1 and it has pro-fibrotic effects including myofibroblast differentiation as described in Figure 3 [165]. Kidney epithelial cells have been reported to release an increased amount of exosomes in response to injury, loaded with specific pro-fibrotic cargo such as TGF-β1 mRNA, which produce fibroblasts proliferation, activation and repair processes [166].

On an IPF rat model, miR-328 was overexpressed in M2 macrophage-derived exosomes with associated proliferative effects in fibroblasts via silencing *FAM13A* and overexpression of certain proteins as collagen 1A and α-SMA (Figure 3) [144]. Three more exosomal microRNAs have been reported to be altered in IS from IPF patients, miR-33a, miR-142 and let-7d [145].

Lastly, a recent study showed increase amount of EVs carrying WNT5A in BALF from IPF patients. These EVs originating from lung fibroblasts have autocrine effects in vitro stimulating fibroblast proliferation [135]. Impairment or block of EV-WNT5A suppresses the fibrogenic effects of EVs improving the IPF pathology.

## 6. EVs in Airway Secretions and Potential Utility in Clinical Practice

Presence of EVs in airway secretions was first described on BALF of healthy subjects [12]. By contrast, the isolation of EVs from sputum was reported 13 years later from spontaneous sputum of cystic fibrosis patients [167]. Following we present the emerging relevance of EVs from airways secretion on LD.

### 6.1. Airway EVs in Asthma

The vast majority of EV studies in patients with asthma have been done using BALF. Increased amount of exosomes with specific protein surface markers [168], lipid [168] and miRNA [139] profiles were found in BALF of asthmatic patients. Exosome concentration and expression of altered EV-miRNAs were correlated with asthma severity biomarkers such as serum eosinophil and IgE levels [168] and FEV_1_ [139]. Up to now, only one work has recovered EVs from IS of asthmatic patients [11]. Exosomes from IS of mild allergic asthma patients contain short RNA species and surface markers as tetraspanins and HLA-DR, suggesting the feasibility of IS as an accessible minimally-invasive source of EV biomarkers [11].

EVs were isolated from NL for the first time on 2011 [169]. More than 600 proteins have been identified among NL-EV cargo, finding proteins related with antimicrobial and barrier functions decreased in patients with asthma and rhinitis [170]. Patients with nasal polyps, commonly associated with asthma, displayed increased levels of ADAM10 in exosomes from NL, which promote angiogenesis and vascular permeability contributing to nasal polyps formation [171].

Asthma is a complex and not fully understood LD. It chronic inflammatory status and remodeling is located in the airway. As we mentioned before, several studies have shown the relationship of EVs from IS, BALF or NL with asthma. Therefore, EVs obtained from the different airway secretions could help to clarify what is happening in the pathology. Those EVs are secreted from the cells involved in the pathogenesis and they have a great potential as both diagnostic tool and therapeutic target. As biomarkers, they can provide a new and more accurate definition of asthma phenotypes, which is crucial for a better treatment. However, it is necessary further analyses that fully characterized both the EVs and their cargo. Specially, of great interest those studies comparing between not only healthy subjects and asthmatic patients but also between different profiles of patients.

In terms of EV-based therapeutics, synthetic liposomes loaded with SOCS3, whose secretion is impaired in asthma, restored the immunomodulatory effects of EVs in an allergic asthma murine model [148]. Finally, MSC-derived EVs have promising immunomodulatory effects in both allergic asthma murine models [172,173] and in vitro cell cultures [173,174]. MSC-derived EVs induce regulatory T cells proliferation on asthmatic patients [174] and inhibit ILC2 activity from allergic rhinitis patients and a murine asthma model [173]. Those results implies their promising future for asthma therapy although extensive studies in vitro and in animal models are required before their translation to the clinic.

### 6.2. Airway EVs in COPD

EVs from airway secretions have also emerged as a potential tool for COPD research, mainly those from BALF. Exosomes from BALF of COPD patients expressed CD66b and NE inducing a COPD phenotype into normal mice [132]. Furthermore, in one study, three EV-miRNAs (let-7e, let-7g and miR-26b) were found altered in BALF of smokers [142]. Furthermore, those smokers BALF-EVs altered human BECs in vitro promoting lung cancer and COPD-like phenotype development [142]. Similarly, 3 exosomal miRNAs from BALF have shown high discriminatory power between COPD patients and healthy ex-smokers [175]. IS-MVs from COPD patients were classified by their cellular origin, being endothelial CD31^+^ MVs and granulocyte CD66b^+^ MVs correlated with COPD severity parameters [133]. Those studies show the importance of airway EVs and their cargo in the progression of COPD, even their ability to promote it phenotype.

COPD is a complex pathology with high impact in the society. Although there is a significant number of studies focus on this pathology, several aspects of it mechanisms and pathophysiology remain unclear. Among them, the heterogeneity of COPD patients is a critical point. Airway EVs, due to their specific cargo, could help to classify the patients depending on their risk to suffer a mainly parenchymal injure, developing an emphysema, and those patients whose main affectation is the bronchial tree, showing chronic bronchitis and the subsequently airway remodeling. Studies that evaluate the cargo of those phenotypes are required for their transalation to the precision medicine.

EVs constitute also a novel therapeutic strategy for COPD. Recently, it has been developed an inhaled formula to target a specific miRNA to lung cells by packaging it into synthetic nanoparticles [176]. Another protocol to transform mice serum derived-EVs with small RNAs were developed and intratracheally administrated in mice with induced lung inflammation. Those EVs were taken up by alveolar macrophages and successfully modulated lung inflammation without trigger immunogenic adverse responses [177]. Those findings set the basis for the development of inhaled EV-based therapies for LD.

### 6.3. Airway EVs in IPF

The interest on the role of EVs in fibrotic diseases have raised during the last years [178]. Two different studies on BALF showed that EVs from IPF patients increase their tissue factor [134] and WNT5A [135] levels. Those tissue factor-bearing MVs were inversely correlated with lung function parameters [134]. In addition, blocking WNT5A reduces the profibrotic effects of those EVs, proposing WNT5A as a potential diagnostic and therapeutic target [135]. In case of IS, a panel of 3 exosomal miRNAs (miR-142-3p, let-7d-5p and miR-33a-5p) allowed to distinguish IPF from healthy subjects. Furthermore, two of them, miR-142-3p and let-7d-5p, were correlated with parameters of IPF severity such as lung diffusion capacity and alveolar-capillary function [145].

Patients with IPF also display a high heterogeneity. While in some of them the disease progresses slowly, others suffer a quick progression with the fatal consequences associated with this pathology. The EVs and their cargo could explain those differences helping to stratify different group of patients and to apply an individually therapy for each subject. Further studies of large population that include different profiles of IPF patients will contribute to those aspects.

EV-based therapeutic approaches for IPF have been barely investigated. EVs from human bone marrow MSCs prevent and alleviate IPF features in a murine model by restoring normal monocyte/macrophage profile, both in vivo and in vitro [179]. By contrast, other studies have focused on antifibrotic effects of specific populations of EVs. Activation of fibroblasts stimulates their EV release carrying PGE_2_, producing inhibition of both TGF-β-induced myofibroblast differentiation and excessive extracellular matrix production on naïve fibroblast [137]. Similarly, MSC-derived EVs also showed to exert aforementioned effects on fibroblasts via their specific miRNA cargo [180]. Although those studies are few, their potential is great due to the absence of an optimal treatment for IPF.

## 7. Conclusions

Asthma, COPD and IPF are multifactorial and complex inflammatory LD which represent an immense health challenging globally. The specific pathways involved in their pathophysiology remains unknown. Airway secretion analysis are included among the routine clinical practice of these diseases; however, their potential is still underestimated despite of their low invasion. EVs could play a crucial role in LD pathogenesis and progression, including asthma, COPD and IPF. Their capacity to transfer information between several cell types involved in LD confers them and their cargo the potential of being not only a novel source of diagnostic and prognostic biomarkers, but also a new therapeutic tool. Despite their potential, some aspects need to be improve before the translation of EV to medical practice. Although the field is evolving fast, the difficulty to stablish an optimal isolation method, the inter-laboratory reproducibility of the results and the high-cost of the equipment are some of these barriers.

The relevance of EVs from airway secretions on LD could be even higher than circulating EVs due to the physiological origin of these diseases. Nevertheless, their technical issues are also more difficult because of the relatively low EV concentration in airway secretions compared to blood. Therefore, further studies are needed to overcome some challenges such as stablish an optimal EVs isolation, fully characterized those EVs or complete reliable profiles of not only EV-miRNAs and EV-proteins but also EV-lncRNA and EV-lipids, specifically in those airway secretions obtained with non-invasive methods as induced sputum or nasal lavage. Additionally, more studies are needed to describe the relationship between the EVs present in different airway secretions, peripheral blood and lung parenchima in the same subject. Therefore, we can clarify the origin and distribution of those EVs and the role they play in the lung and the lower airway. Altogether, those studies may provide novel specific and minimally non-invasive biomarkers, which would surely improve the clinical management of LD.

## Figures and Tables

**Table 1:** Proteins, lipids and miRNAs contained in extracellular vesicles (EVs) that have been associated with asthma, chronic obstructive pulmonary disease (COPD) and idiopathic pulmonary fibrosis (IPF).

EV Cargo	Asthma	COPD	IPF
Proteins	ADAM10 [11], MHC-II [101,103,130], HLA-DR (IS) [11]	α1-antitrypsin [131], CD66b [132,133], CD31, NE [132]	Tissue factor [134], WNT5A [135]
Lipids	Leukotriene B4 [136]		PGE2 [137]
miRNAs	miR-223 and miR-142a [138], let-7, miR-200 families, miR-21 [139]	miR-210 [140], miR-21 [141], Let-7e, let-7g, miR-26b [142]	miR-21 [143], miR-328 [144], miR-33a, miR-142 and let-7d [145]

## Data Availability

Not applicable.

## Abbreviations

α-SMA	Alpha-smooth muscle actin
AEC1	Alveolar epithelial cell 1
AEC2	Alveolar epithelial cell 2
BECs	Bronchial epithelial cells
BALF	Bronchoalveolar lavage fluid
COPD	Chronic obstructive pulmonary disease
CCN	CYR61/CTGF/NOV protein family
CCL	C-C motif chemokine ligand
CXCL	C-X-C motif chemokine ligand
CS	Cigarette smoke
EV	Extracellular vesicle
FEV_1_	Forced expiratory volume in one second
FVC	Forced vital capacity
IPF	Idiopathic pulmonary fibrosis
IS	Induced sputum
ICS	Inhaled corticosteroids
ICAM-1	Intercellular adhesion molecule 1
LD	Lung Diseases
MMP	Matrix metallopeptidase
MSC	Mesenchymal stem cell
MCP-1	Monocyte chemoattractant protein 1
mRNA	Messenger RNA
miRNA	MicroRNA
MVBs	Multivesicular bodies
NL	Nasal lavage fluid
NO	Nitric oxide
NF-κβ	Nuclear factor kappa beta
OSA	Obstructive sleep apnea
PHAL	Pharyngeal lavage fluid
PGE_2_	Prostaglandin E2
ROS	Reactive oxygen species
SASP	Senescence-associated secretory phenotype
SOCS	Suppressor of cytokines signaling
TNF-α	Tumoral necrosis factor alpha
TGF- β	Transforming growth factor beta
WNT	Wingless-type mouse mammary tumor virus integration site family

## References

[1] European Respiratory Society (2017). The Global Impact of Respiratory Disease.

[2] Shaykhiev R., Otaki F., Bonsu P., Dang D.T., Teater M., Strulovici-Barel Y., Salit J., Harvey B.-G., Worgall S. (2011). Cigarette smoking reprograms apical junctional complex molecular architecture in the human airway epithelium in vivo. Cell. Mol. Life Sci..

[3] Andres J., Smith L.C., Murray A., Jin Y., Businaro R., Laskin J.D., Laskin D.L. (2020). Role of extracellular vesicles in cell-cell communication and inflammation following exposure to pulmonary toxicants. Cytokine Growth Factor Rev..

[4] Schraufnagel D.E., Balmes J.R., Cowl C.T., De Matteis S., Jung S.-H., Mortimer K., Perez-Padilla R., Rice M.B., Riojas-Rodriguez H., Sood A. (2019). Air Pollution and Noncommunicable Diseases A Review by the Forum of International Respiratory Societies’ Environmental Committee, Part 2: Air Pollution and Organ Systems. Chest.

[5] Barnes P.J. (2017). Cellular and molecular mechanisms of asthma and COPD. Clin. Sci..

[6] Muller L., Hong C.-S., Stolz D.B., Watkins S.C., Whiteside T.L. (2014). Isolation of biologically-active exosomes from human plasma. J. Immunol. Methods.

[7] Serrano-Pertierra E., Oliveira-Rodríguez M., Rivas M., Oliva P., Villafani J., Navarro A., Blanco-López M.C., Cernuda-Morollón E. (2019). Characterization of Plasma-Derived Extracellular Vesicles Isolated by Different Methods: A Comparison Study. Bioengineering.

[8] Arraud N., Linares R., Tan S., Gounou C., Pasquet J.-M., Mornet S., Brisson A.R. (2014). Extracellular vesicles from blood plasma: Determination of their morphology, size, phenotype and concentration. J. Thromb. Haemost..

[9] Crossland R.E., Norden J., Bibby L.A., Davis J., Dickinson A.M. (2016). Evaluation of optimal extracellular vesicle small RNA isolation and qRT-PCR normalisation for serum and urine. J. Immunol. Methods.

[10] Lässer C., Alikhani V.S., Ekström K., Eldh M., Paredes P.T., Bossios A., Sjöstrand M., Gabrielsson S., Lötvall J., Valadi H. (2011). Human saliva, plasma and breast milk exosomes contain RNA: Uptake by macrophages. J. Transl. Med..

[11] Sánchez-Vidaurre S., Eldh M., Larssen P., Daham K., Martinez-Bravo M.-J., Dahlén S.-E., Dahlén B., Van Hage M., Gabrielsson S. (2017). RNA-containing exosomes in induced sputum of asthmatic patients. J. Allergy Clin. Immunol..

[12] Admyre C., Grunewald J., Thyberg J., Gripenbäck S., Tornling G., Eklund A., Scheynius A., Gabrielsson S. (2003). Exosomes with major histocompatibility complex class II and co-stimulatory molecules are present in human BAL fluid. Eur. Respir. J..

[13] Van Niel G., D’Angelo G., Raposo G. (2018). Shedding light on the cell biology of extracellular vesicles. Nat. Rev. Mol. Cell Biol..

[14] Kalluri R., LeBleu V.S. (2020). The biology, function, and biomedical applications of exosomes. Science.

[15] Lee H., Zhang D., Zhu Z., Cruz C.S.D., Jin Y. (2016). Epithelial cell-derived microvesicles activate macrophages and promote inflammation via microvesicle-containing microRNAs. Sci. Rep..

[16] Moon H.-G., Cao Y., Yang J., Lee J.H., Choi H.S., Jin Y. (2015). Lung epithelial cell-derived extracellular vesicles activate macrophage-mediated inflammatory responses via ROCK1 pathway. Cell Death Dis..

[17] Stremersch S., De Smedt S.C., Raemdonck K. (2016). Therapeutic and diagnostic applications of extracellular vesicles. J. Control. Release.

[18] Clemmens H., Lambert D.W. (2018). Extracellular vesicles: Translational challenges and opportunities. Biochem. Soc. Trans..

[19] Global Asthma Network (2018). The Global Asthma Report.

[20] Ray A., Oriss T.B., Wenzel S.E. (2015). Emerging molecular phenotypes of asthma. Am. J. Physiol. Cell. Mol. Physiol..

[21] Wenzel S.E. (2016). Emergence of Biomolecular Pathways to Define Novel Asthma Phenotypes. Type-2 Immunity and Beyond. Am. J. Respir. Cell Mol. Biol..

[22] Upham J.W., Xi Y. (2017). Dendritic Cells in Human Lung Disease: Recent Advances. Chest.

[23] Takhar P., Corrigan C.J., Smurthwaite L., O’Connor B.J., Durham S.R., Lee T.H., Gould H.J. (2007). Class switch recombination to IgE in the bronchial mucosa of atopic and nonatopic patients with asthma. J. Allergy Clin. Immunol..

[24] Bousquet J., Chanez P., Lacoste J.Y., Barnéon G., Ghavanian N., Enander I., Venge P., Ahlstedt S., Simony-Lafontaine J., Godard P. (1990). Eosinophilic Inflammation in Asthma. N. Engl. J. Med..

[25] Kuruvilla M.E., Lee F.E.-H., Lee G. (2019). Understanding Asthma Phenotypes, Endotypes, and Mechanisms of Disease. Clin. Rev. Allergy Immunol..

[26] Al-Ramli W., Préfontaine D., Chouiali F., Martin J.G., Olivenstein R., Lemiere C., Hamid Q. (2009). TH17-associated cytokines (IL-17A and IL-17F) in severe asthma. J. Allergy Clin. Immunol..

[27] Boucherat O., Boczkowski J., Jeannotte L., Delacourt C. (2013). Cellular and molecular mechanisms of goblet cell metaplasia in the respiratory airways. Exp. Lung Res..

[28] Black J.L., Panettieri R.A., Banerjee A., Berger P. (2012). Airway Smooth Muscle in Asthma. Just a Target for Bronchodilation?. Clin. Chest Med..

[29] Burney P.G., Patel J., Newson R., Minelli C., Naghavi M. (2015). Global and regional trends in COPD mortality, 1990–2010. Eur. Respir. J..

[30] (2020). Global Strategy for the Diagnosis, Management, and Prevention of Chronic Obstructive Pulmonary Disease, Global Initiative for Chronic Obstructive Lung Disease (GOLD), USA. www.goldcopd.org.

[31] Gan W.Q., Man S.F.P., Senthilselvan A., Sin D.D. (2004). Association between chronic obstructive pulmonary disease and systemic inflammation: A systematic review and a meta-analysis. Thorax.

[32] Mercado N., Ito K., Barnes P.J. (2015). Accelerated ageing of the lung in COPD: New concepts. Thorax.

[33] McDonough J.E., Yuan R., Suzuki M., Seyednejad N., Elliott W.M., Sanchez P.G., Wright A.C., Gefter W.B., Litzky L., Coxson H.O. (2011). Small-Airway Obstruction and Emphysema in Chronic Obstructive Pulmonary Disease. N. Engl. J. Med..

[34] Barnes P.J. (2014). Cellular and Molecular Mechanisms of Chronic Obstructive Pulmonary Disease. Clin. Chest Med..

[35] Caramori G., Romagnoli M., Casolari P., Bellettato C., Casoni G., Boschetto P., Chung K.F., Barnes P.J., Adcock I.M., Ciaccia A. (2003). Nuclear localisation of p65 in sputum macrophages but not in sputum neutrophils during COPD exacerbations. Thorax.

[36] Russell R., Thorley A., Culpitt S.V., Dodd S., E Donnelly L., DeMattos C., Fitzgerald M., Barnes P.J. (2002). Alveolar macrophage-mediated elastolysis: Roles of matrix metalloproteinases, cysteine, and serine proteases. Am. J. Physiol. Cell. Mol. Physiol..

[37] Hodge S., Hodge G., Scicchitano R., Reynolds P.N., Holmes M. (2003). Alveolar macrophages from subjects with chronic obstructive pulmonary disease are deficient in their ability to phagocytose apoptotic airway epithelial cells. Immunol. Cell Biol..

[38] Bafadhel M., Saha S.K., Siva R., McCormick M., Monteiro W., Rugman P., Dodson P., Pavord I.D., Newbold P., Brightling C.E. (2009). Sputum IL-5 Concentration Is Associated with a Sputum Eosinophilia and Attenuated by Corticosteroid Therapy in COPD. Respiration.

[39] Hutchinson J.P., Fogarty A.W., Hubbard R.B., McKeever T.M. (2015). Global incidence and mortality of idiopathic pulmonary fibrosis: A systematic review. Eur. Respir. J..

[40] Raghu G., Weycker D., Edelsberg J., Bradford W.Z., Oster G. (2006). Incidence and Prevalence of Idiopathic Pulmonary Fibrosis. Am. J. Respir. Crit. Care Med..

[41] Ley B., Collard H.R., King T.E. (2011). Clinical Course and Prediction of Survival in Idiopathic Pulmonary Fibrosis. Am. J. Respir. Crit. Care Med..

[42] Kasper M., Haroske G. (1996). Alterations in the alveolar epithelium after injury leading to pulmonary fibrosis. Histol. Histopathol..

[43] Liang J., Zhang Y., Xie T., Liu N., Chen H., Geng Y., Kurkciyan A., Mena J.M., Stripp B.R., Jiang D. (2016). Hyaluronan and TLR4 promote surfactant-protein-C-positive alveolar progenitor cell renewal and prevent severe pulmonary fibrosis in mice. Nat. Med..

[44] Selman M., King T.E., Pardo A. (2001). Idiopathic Pulmonary Fibrosis: Prevailing and Evolving Hypotheses about Its Pathogenesis and Implications for Therapy. Ann. Intern. Med..

[45] Rock J.R., Barkauskas C.E., Cronce M.J., Xue Y., Harris J.R., Liang J., Noble P.W., Hogan B.L. (2011). Multiple stromal populations contribute to pulmonary fibrosis without evidence for epithelial to mesenchymal transition. Proc. Natl. Acad. Sci. USA.

[46] Horowitz J.C., Thannickal V.J. (2006). Epithelial-Mesenchymal Interactions in Pulmonary Fibrosis. Semin. Respir. Crit. Care Med..

[47] Hung C., Linn G., Chow Y.-H., Kobayashi A., Mittelsteadt K., Altemeier W.A., Gharib S.A., Schnapp L.M., Duffield J.S. (2013). Role of Lung Pericytes and Resident Fibroblasts in the Pathogenesis of Pulmonary Fibrosis. Am. J. Respir. Crit. Care Med..

[48] Liu F., Mih J.D., Shea B.S., Kho A.T., Sharif A.S., Tager A.M., Tschumperlin D.J. (2010). Feedback amplification of fibrosis through matrix stiffening and COX-2 suppression. J. Cell Biol..

[49] Boers J.E., Ambergen A.W., Thunnissen F.B.J.M. (1998). Number and Proliferation of Basal and Parabasal Cells in Normal Human Airway Epithelium. Am. J. Respir. Crit. Care Med..

[50] Hong K.U., Reynolds S.D., Watkins S., Fuchs E., Stripp B.R. (2004). Basal Cells Are a Multipotent Progenitor Capable of Renewing the Bronchial Epithelium. Am. J. Pathol..

[51] Chilosi M., Poletti V., Murer B., Lestani M., Cancellieri A., Montagna L., Piccoli P., Cangi G., Semenzato G., Doglioni C. (2002). Abnormal Re-epithelialization and Lung Remodeling in Idiopathic Pulmonary Fibrosis: The Role of ΔN-p63. Lab. Investig..

[52] Costabel U., Danel C., Haslam P., Higgenbottam T., Klech H., Pohl W., Semenzato G. (1989). Technical Recommendations and Guidelines for Bronchoalveolar Lavage (BAL). Report of the European Society of Pneumology Task Group. Eur. Respir. J..

[53] Meyer K.C., Raghu G., Baughman R.P., Brown K.K., Costabel U., Du Bois R.M., Drent M., Haslam P.L., Kim D.S., Nagai S. (2012). An Official American Thoracic Society Clinical Practice Guideline: The Clinical Utility of Bronchoalveolar Lavage Cellular Analysis in Interstitial Lung Disease. Am. J. Respir. Crit. Care Med..

[54] Baughman R.P. (2007). Technical Aspects of Bronchoalveolar Lavage: Recommendations for a Standard Procedure. Semin. Respir. Crit. Care Med..

[55] Wattiez R., Hermans C., Cruyt C., Bernard A., Falmagne P. (2000). Human Bronchoalveolar Lavage Fluid Protein Two-Dimensional Database: Study of Interstitial Lung Diseases. Electrophoresis.

[56] Conickx G., Cobos F.A., Berge M.V.D., Faiz A., Timens W., Hiemstra P.S., Joos G.F., Brusselle G., Mestdagh P., Bracke K. (2017). microRNA profiling in lung tissue and bronchoalveolar lavage of cigarette smoke-exposed mice and in COPD patients: A translational approach. Sci. Rep..

[57] Van Der Vliet A., O’Neill C.A., Cross C.E., Koostra J.M., Volz W.G., Halliwell B., Louie S. (1999). Determination of low-molecular-mass antioxidant concentrations in human respiratory tract lining fluids. Am. J. Physiol. Cell. Mol. Physiol..

[58] Radha S., Ravindra N., Afroz T., Prasad S. (2014). Diagnostic utility of bronchoalveolar lavage. J. Cytol..

[59] Meyer K.C. (2007). Bronchoalveolar Lavage as a Diagnostic Tool. Semin. Respir. Crit. Care Med..

[60] Guiot J., Demarche S.F., Henket M., Paulus V., Graff S., Schleich F., Corhay J.-L., Louis R., Moermans C. (2017). Methodology for Sputum Induction and Laboratory Processing. J. Vis. Exp..

[61] Paggiaro P.L., Chanez P., Holz O., Ind P.W., Djukanović R., Maestrelli P., Sterk P.J. (2002). Sputum Induction. In European Respiratory Journal, Supplement. Eur. Respir. Soc..

[62] Pizzichini M.M.M., Leigh R., Djukanovic R., Sterk P.J. (2002). Safety of Sputum Induction. Eur. Respir. J..

[63] Antoniou K.M., Alexandrakis M., Tzanakis N., Tsiligianni I., Tzortzaki E.G., Siafakas N.M., Bouros D. (2005). Induced Sputum versus Bronchoalveolar Lavage Fluid in the Evaluation of Patients with Idiopathic Pulmonary Fibrosis. Respiration.

[64] Efthimiadis A., Spanevello A., Hamid Q., Kelly M.M., Linden M., Louis R., Pizzichini M.M.M., Pizzichini E., Ronchi C., Van Overveld F. (2002). Methods of Sputum Processing for Cell Counts, Immunocytochemistry and in Situ Hybridisation. Eur. Respir. J..

[65] Belda J., Leigh R., Parameswaran K., O’Byrne P.M., Sears M.R., Hargreave F.E. (2000). Induced Sputum Cell Counts in Healthy Adults. Am. J. Respir. Crit. Care Med..

[66] Brightling C. (2006). Clinical Applications of Induced Sputum. Chest.

[67] Schleich F., Manise M., Sele J., Henket M., Seidel L., Louis R. (2013). Distribution of sputum cellular phenotype in a large asthma cohort: Predicting factors for eosinophilic vs neutrophilic inflammation. BMC Pulm. Med..

[68] Seys S.F. (2017). Role of sputum biomarkers in the management of asthma. Curr. Opin. Pulm. Med..

[69] Haldar P., Pavord I.D. (2007). Noneosinophilic asthma: A distinct clinical and pathologic phenotype. J. Allergy Clin. Immunol..

[70] Simpson J.L., Scott R., Boyle M.J., Gibson P.G. (2006). Inflammatory subtypes in asthma: Assessment and identification using induced sputum. Respirology.

[71] Global Strategy for Asthma Management and Prevention Updated 2020. www.ginasthma.org.

[72] Vogelmeier C., Criner G.J., Martinez F.J., Anzueto A., Barnes P.J., Bourbeau J., Celli B.R., Chen R., Decramer M., Fabbri L.M. (2017). Global Strategy for the Diagnosis, Management, and Prevention of Chronic Obstructive Lung Disease 2017 Report. GOLD Executive Summary. Am. J. Respir. Crit. Care Med..

[73] D’Silva L., Cook R.J., Allen C.J., Hargreave F.E., Parameswaran K. (2007). Changing pattern of sputum cell counts during successive exacerbations of airway disease. Respir. Med..

[74] Sobiecka M., Kus J., Demkow U., Filewska M., Jozwik A., Radwan-Rohrenschef P., Chorostowska-Wynimko J. (2008). Induced sputum in patients with interstitial lung disease: A non-invasive surrogate for certain parameters in bronchoalveolar lavage fluid. J. Physiol. Pharmacol..

[75] Belda J., Parameswaran K., Keith P.K., Hargreave F.E. (2001). Repeatability and validity of cell and fluid-phase measurements in nasal fluid: A comparison of two methods of nasal lavage. Clin. Exp. Allergy.

[76] Greiff L., Pipkorn U., Alkner U., Persson C.G.A. (1990). The’nasal pool’device applies controlled concentrations of solutes on human nasal airway mucosa and samples its surface exudations/secretions. Clin. Exp. Allergy.

[77] Grünberg K., Timmers M.C., Smits H.H., de Klerk E.P., Dick E.C., Spaan W.J., Hiemstra P.S., Sterk P.J. (1997). Effect of Experimental Rhinovirus 16 Colds on Airway Hyperresponsiveness to Histamine and Interleukin-8 in Nasal Lavage in Asthmatic Subjects In Vivo. Clin. Exp. Allergy.

[78] Prat J., Xaubet A., Mullol J., Plaza V., Masó M., Lleonart R., Picado C. (1993). Immunocytologic analysis of nasal cells obtained by nasal lavage: A comparative study with a standard method of cell identification. Allergy.

[79] Zhang Y., Chen W., Ji J.F., Wang Z.Y., Wu M.H., Cheng Y., Jiang M.J., Wang Q.P., Chen R.J. (2019). The significance of eosinophils in the correlation of upper and lower airway inflammation in patients with chronic rhinitis. Zhonghua Er Bi Yan Hou Tou Jing Wai Ke Za Zhi.

[80] Simpson J.L., Bafadhel M. (2017). Alternatives to induced sputum for identifying inflammatory subtypes of asthma. Respirology.

[81] De Farias C.F., Amorim M.M., Dracoulakis M., Caetano L.B., Santoro I.L., Fernandes A.L.G. (2016). Nasal lavage, blood or sputum: Which is best for phenotyping asthma?. Respirology.

[82] Celik H., Akpinar S., Karabulut H., Oktar P., Dursun B., Erguden H.C., Gunay S., Sipit T. (2014). Evaluation of IL-8 Nasal Lavage Levels and the Effects of Nasal Involvement on Disease Severity in Patients with Stable Chronic Obstructive Pulmonary Disease. Inflammation.

[83] Hauber H.P., Rüller S., Müller Peter E., Peter Z. (2009). Comparison of Differential Cell Counts in Pharyngeal Lavage from Patients with Obstructive Sleep Apnea and from Healthy Control Persons. Atemwegs- und Lungenkrankheiten.

[84] Vicente E., Marin J.M., Carrizo S.J., Osuna C.S., González R., Marin-Oto M., Forner M., Vicente P., Cubero P., Gil A.V. (2016). Upper airway and systemic inflammation in obstructive sleep apnoea. Eur. Respir. J..

[85] I Baess A., E Mohamed E., Eldowik Y.M. (2019). Study of upper airway inflammation in patients with obstructive sleep apnea–hypopnea syndrome. Egypt. J. Bronchol..

[86] Hauber H.-P., Rüller S., Müller E., Hansen E., Zabel P. (2011). Pharyngeal Lavage Lymphocytosis in Patients with Obstructive Sleep Apnea: A Preliminary Observation. PLoS ONE.

[87] Théry C., Witwer K.W., Aikawa E., Alcaraz M.J., Anderson J.D., Andriantsitohaina R., Antoniou A., Arab T., Archer F., Atkin-Smith G.K. (2018). Minimal information for studies of extracellular vesicles 2018 (MISEV2018): A position statement of the International Society for Extracellular Vesicles and update of the MISEV2014 guidelines. J. Extracell. Vesicles.

[88] György B., Szabó T.G., Pásztói M., Pál Z., Misják P., Aradi B., László V., Pállinger É., Pap E., Kittel Á. (2011). Membrane vesicles, current state-of-the-art: Emerging role of extracellular vesicles. Cell. Mol. Life Sci..

[89] Pathan M., Fonseka P., Chitti S.V., Kang T., Sanwlani R., Van Deun J., Hendrix A., Mathivanan S. (2019). Vesiclepedia 2019: A compendium of RNA, proteins, lipids and metabolites in extracellular vesicles. Nucleic Acids Res..

[90] Mathieu M., Martin-Jaular L., Lavieu G., Théry C. (2019). Specificities of secretion and uptake of exosomes and other extracellular vesicles for cell-to-cell communication. Nat. Cell Biol..

[91] Johnstone R.M., Adam M., Hammond J.R., Orr L., Turbide C. (1987). Vesicle formation during reticulocyte maturation. Association of plasma membrane activities with released vesicles (exosomes). J. Biol. Chem..

[92] Kowal J., Tkach M., Théry C. (2014). Biogenesis and secretion of exosomes. Curr. Opin. Cell Biol..

[93] Heijnen H.F., Schiel A.E., Fijnheer R., Geuze H.J., Sixma J.J. (1999). Activated Platelets Release Two Types of Membrane Vesicles: Microvesicles by Surface Shedding and Exosomes Derived From Exocytosis of Multivesicular Bodies and -Granules. Blood.

[94] Cocucci E., Meldolesi J. (2015). Ectosomes and exosomes: Shedding the confusion between extracellular vesicles. Trends Cell Biol..

[95] Kerr J.F.R., Wyllie A.H., Currie A.R. (1972). Apoptosis: A Basic Biological Phenomenon with Wideranging Implications in Tissue Kinetics. Br. J. Cancer.

[96] Atkin-Smith G.K., Tixeira R., Paone S., Mathivanan S., Collins C., Liem M., Goodall K.J., Ravichandran K.S., Hulett M.D., Poon I.K.H. (2015). A novel mechanism of generating extracellular vesicles during apoptosis via a beads-on-a-string membrane structure. Nat. Commun..

[97] Wubbolts R., Leckie R.S., Veenhuizen P.T.M., Schwarzmann G., Möbius W., Hoernschemeyer J., Slot J.W., Geuze H.J., Stoorvogel W. (2003). Proteomic and Biochemical Analyses of Human B Cell-Derived Exosomes: Potential Implications for Their Function and Multivesicular Body Formation. J. Biol. Chem..

[98] Crescitelli R., Lässer C., Szabó T.G., Kittel A., Eldh M., Dianzani I., Buzás E.I., Lotvall J. (2013). Distinct RNA profiles in subpopulations of extracellular vesicles: Apoptotic bodies, microvesicles and exosomes. J. Extracell. Vesicles.

[99] Anand S., Samuel M., Kumar S., Mathivanan S. (2019). Ticket to a bubble ride: Cargo sorting into exosomes and extracellular vesicles. Biochim. Biophys. Acta (BBA)—Proteins Proteom..

[100] Wolfers J., Lozier A., Raposo G., Regnault A., Théry C., Masurier C., Flament C., Pouzieux S., Faure F., Tursz T. (2001). Tumor-derived exosomes are a source of shared tumor rejection antigens for CTL cross-priming. Nat. Med..

[101] Vallhov H., Gutzeit C., Hultenby K., Valenta R., Gronlund H., Scheynius A. (2015). Dendritic cell-derived exosomes carry the major cat allergen Fel d 1 and induce an allergic immune response. Allergy.

[102] Blanchard N., Lankar D., Faure F., Regnault A., Dumont C., Raposo G., Hivroz C. (2002). TCR Activation of Human T Cells Induces the Production of Exosomes Bearing the TCR/CD3/ζ Complex. J. Immunol..

[103] Raposo G., Nijman H.W., Stoorvogel W., Liejendekker R., Harding C.V., Melief C.J., Geuze H.J. (1996). B lymphocytes secrete antigen-presenting vesicles. J. Exp. Med..

[104] Mazzeo C., Cañas J.A., Zafra M.P., Mar F.-N., Fernández-Nieto M., Sanz V., Mittelbrunn M., Izquierdo M., Baixaulli F., Sastre J. (2015). Exosome secretion by eosinophils: A possible role in asthma pathogenesis. J. Allergy Clin. Immunol..

[105] Hristov M., Erl W., Linder S., Weber P.C. (2004). Apoptotic bodies from endothelial cells enhance the number and initiate the differentiation of human endothelial progenitor cells in vitro. Blood.

[106] Qiu G., Zheng G., Ge M., Wang J., Huang R., Shu Q., Xu J. (2018). Mesenchymal stem cell-derived extracellular vesicles affect disease outcomes via transfer of microRNAs. Stem Cell Res. Ther..

[107] Riancho J., Sánchez-Juan P. (2018). Circulating Extracellular Vesicles in Human Disease. N. Engl. J. Med..

[108] De Toro J., Herschlik L., Waldner C., Mongini C. (2015). Emerging Roles of Exosomes in Normal and Pathological Conditions: New Insights for Diagnosis and Therapeutic Applications. Front. Immunol..

[109] Chimen M., Evryviadou A., Box C.L., Harrison M.J., Hazeldine J., Dib L.H., Kuravi S.J., Payne H., Price J.M., Kavanagh D. (2019). Appropriation of GPIbα from platelet-derived extracellular vesicles supports monocyte recruitment in systemic inflammation. Haematologica.

[110] Zulueta A., Peli V., Cas M.D., Colombo M., Paroni R., Falleni M., Baisi A., Bollati V., Chiaramonte R., Del Favero E. (2019). Inflammatory role of extracellular sphingolipids in Cystic Fibrosis. Int. J. Biochem. Cell Biol..

[111] Wadey R.M., Connolly K.D., Mathew D., Walters G., Rees D.A., E James P. (2019). Inflammatory adipocyte-derived extracellular vesicles promote leukocyte attachment to vascular endothelial cells. Atherosclerosis.

[112] Goetzl E.J., Schwartz J.B., Mustapic M., Lobach I.V., Daneman R., Abner E.L., Jicha G.A. (2017). Altered cargo proteins of human plasma endothelial cell–derived exosomes in atherosclerotic cerebrovascular disease. FASEB J..

[113] Chang W., Wang J. (2019). Exosomes and Their Noncoding RNA Cargo Are Emerging as New Modulators for Diabetes Mellitus. Cells.

[114] Li F., Li H., Jin X., Zhang Y., Kang X., Zhang Z., Xu M., Qian Z., Ma Z., Gao X. (2019). Adipose-specific knockdown of Sirt1 results in obesity and insulin resistance by promoting exosomes release. Cell Cycle.

[115] Bell E., A Taylor M. (2017). Functional Roles for Exosomal MicroRNAs in the Tumour Microenvironment. Comput. Struct. Biotechnol. J..

[116] Steinbichler T.B., Dudás J., Riechelmann H., Skvortsova I.-I. (2017). The role of exosomes in cancer metastasis. Semin. Cancer Biol..

[117] Chen J., Hu C., Pan P. (2017). Extracellular Vesicle MicroRNA Transfer in Lung Diseases. Front. Physiol..

[118] Kubo H. (2018). Extracellular Vesicles in Lung Disease. Chest.

[119] Lässer C., Jang S.C., Lötvall J. (2018). Subpopulations of extracellular vesicles and their therapeutic potential. Mol. Asp. Med..

[120] Sancho-Albero M., Rubio-Ruiz B., Pérez-López A.M., Sebastian V., Martín-Duque P., Arruebo M., Santamaria J., Unciti-Broceta A. (2019). Cancer-derived exosomes loaded with ultrathin palladium nanosheets for targeted bioorthogonal catalysis. Nat. Catal..

[121] Kan S., Hariyadi D.M., Grainge C., Knight D., Bartlett N.W., Liang M. (2020). Airway epithelial-targeted nanoparticles for asthma therapy. Am. J. Physiol. Cell. Mol. Physiol..

[122] Sanz-Rubio D., Martín-Burriel I., Gil A., Cubero P., Forner M., Khalyfa A., Marin J.M. (2018). Stability of Circulating Exosomal miRNAs in Healthy Subjects. Sci. Rep..

[123] Taylor D.D., Shah S. (2015). Methods of isolating extracellular vesicles impact down-stream analyses of their cargoes. Methods.

[124] Patel G.K., Khan M.A., Zubair H., Srivastava S.K., Khushman M., Singh S., Singh A.P. (2019). Comparative analysis of exosome isolation methods using culture supernatant for optimum yield, purity and downstream applications. Sci. Rep..

[125] Lötvall J., Hill A.F., Hochberg F., Buzás E.I., Di Vizio D., Gardiner C., Gho Y.S., Kurochkin I.V., Mathivanan S., Quesenberry P. (2014). Minimal Experimental Requirements for Definition of Extracellular Vesicles and their Functions: A Position Statement from the International Society for Extracellular Vesicles. J. Extracell. Vesicles.

[126] Kulshreshtha A., Ahmad T., Agrawal A., Ghosh B. (2013). Proinflammatory role of epithelial cell–derived exosomes in allergic airway inflammation. J. Allergy Clin. Immunol..

[127] Gupta R., Radicioni G., Abdelwahab S., Dang H., Carpenter J., Chua M., Mieczkowski P.A., Sheridan J.T., Randell S.H., Kesimer M. (2019). Intercellular Communication between Airway Epithelial Cells Is Mediated by Exosome-Like Vesicles. Am. J. Respir. Cell Mol. Biol..

[128] Kesimer M., Scull M., Brighton B., DeMaria G., Burns K., O’Neal W., Pickles R.J., Sheehan J.K. (2009). Characterization of exosome-like vesicles released from human tracheobronchial ciliated epithelium: A possible role in innate defense. FASEB J..

[129] Bourdonnay E., Zasłona Z., Penke L.R.K., Speth J.M., Schneider D.J., Przybranowski S., Swanson J.A., Mancuso P., Freeman C.M., Curtis J.L. (2015). Transcellular delivery of vesicular SOCS proteins from macrophages to epithelial cells blunts inflammatory signaling. J. Exp. Med..

[130] Admyre C., Bohle B., Johansson S.M., Focke-Tejkl M., Valenta R., Scheynius A., Gabrielsson S. (2007). B cell–derived exosomes can present allergen peptides and activate allergen-specific T cells to proliferate and produce TH2-like cytokines. J. Allergy Clin. Immunol..

[131] Lockett A.D., Brown M.B., Santos-Falcon N., Rush N.I., Oueini H., Oberle A.J., Bolanis E., Fragoso M.A., Petrusca D.N., Serban K.A. (2014). Active Trafficking of Alpha 1 Antitrypsin across the Lung Endothelium. PLoS ONE.

[132] Genschmer K.R., Russell D.W., Lal C., Szul T., Bratcher P.E., Noerager B.D., Roda M.A., Xu X., Rezonzew G., Viera L. (2019). Activated PMN Exosomes: Pathogenic Entities Causing Matrix Destruction and Disease in the Lung. Cell.

[133] Porro C., Lacedonia D., Carpagnano G.E., Trotta T., Palladino G.P., Panaro M.A., Zoppo L.D., Foschino M.P. (2016). Microparticles in sputum of COPD patients: A potential biomarker of the disease?. Int. J. Chronic Obstr. Pulm. Dis..

[134] Novelli F., Neri T., Tavanti L., Armani C., Noce C., Falaschi F., Bartoli M.L., Martino F., Palla A., Celi A. (2014). Procoagulant, Tissue Factor-Bearing Microparticles in Bronchoalveolar Lavage of Interstitial Lung Disease Patients: An Observational Study. PLoS ONE.

[135] Martin-Medina A., Lehmann M., Burgy O., Hermann S., Baarsma H.A., Wagner D.E., De Santis M.M., Ciolek F., Hofer T.P., Frankenberger M. (2018). Increased Extracellular Vesicles Mediate WNT5A Signaling in Idiopathic Pulmonary Fibrosis. Am. J. Respir. Crit. Care Med..

[136] Majumdar R., Tameh A.T., Parent C.A. (2016). Exosomes Mediate LTB4 Release during Neutrophil Chemotaxis. PLoS Biol..

[137] Lacy S.H., Woeller C.F., Thatcher T.H., Pollock S.J., Small E.M., Sime P.J., Phipps R.P. (2019). Activated Human Lung Fibroblasts Produce Extracellular Vesicles with Antifibrotic Prostaglandins. Am. J. Respir. Cell Mol. Biol..

[138] Pua H.H., Happ H.C., Gray C.J., Mar D.J., Chiou N.-T., Hesse L.E., Ansel K.M. (2019). Increased Hematopoietic Extracellular RNAs and Vesicles in the Lung during Allergic Airway Responses. Cell Rep..

[139] Levänen B., Bhakta N.R., Paredes P.T., Barbeau R., Hiltbrunner S., Pollack J.L., Sköld C.M., Svartengren M., Grunewald J., Gabrielsson S. (2013). Altered microRNA profiles in bronchoalveolar lavage fluid exosomes in asthmatic patients. J. Allergy Clin. Immunol..

[140] Fujita Y., Araya J., Ito S., Kobayashi K., Kosaka N., Yoshioka Y., Kadota T., Hara H., Kuwano K., Ochiya T. (2015). Suppression of autophagy by extracellular vesicles promotes myofibroblast differentiation in COPD pathogenesis. J. Extracell. Vesicles.

[141] Xu H., Ling M., Xue J., Dai X., Sun Q., Chen C., Liu Y., Zhou L., Liu J., Luo F. (2018). Exosomal microRNA-21 derived from bronchial epithelial cells is involved in aberrant epithelium-fibroblast cross-talk in COPD induced by cigarette smoking. Theranostics.

[142] Héliot A., Landkocz Y., Saint-Georges F.R., Gosset P., Billet S., Shirali P., Courcot D., Martin P.J. (2017). Smoker extracellular vesicles influence status of human bronchial epithelial cells. Int. J. Hyg. Environ. Heal..

[143] Makiguchi T., Yamada M., Yoshioka Y., Sugiura H., Koarai A., Chiba S., Fujino N., Tojo Y., Ota C., Kubo H. (2016). Serum extracellular vesicular miR-21-5p is a predictor of the prognosis in idiopathic pulmonary fibrosis. Respir. Res..

[144] Yao M.-Y., Zhang W.-H., Ma W.-T., Liu Q.-H., Xing L.-H., Zhao G.-F. (2019). microRNA-328 in exosomes derived from M2 macrophages exerts a promotive effect on the progression of pulmonary fibrosis via FAM13A in a rat model. Exp. Mol. Med..

[145] Njock M.-S., Guiot J., A Henket M., Nivelles O., Thiry M., Dequiedt F., Corhay J.-L., E Louis R., Struman I. (2018). Sputum exosomes: Promising biomarkers for idiopathic pulmonary fibrosis. Thorax.

[146] Park J.-A., Sharif A.S., Tschumperlin D.J., Lau L., Limbrey R., Howarth P., Drazen J.M. (2012). Tissue factor–bearing exosome secretion from human mechanically stimulated bronchial epithelial cells in vitro and in vivo. J. Allergy Clin. Immunol..

[147] Najrana T., Mahadeo A., Abu-Eid R., Kreienberg E., Schulte V., Uzun A., Schorl C., Goldberg L., Quesenberry P., Sanchez-Esteban J. (2020). Mechanical stretch regulates the expression of specific miRNA in extracellular vesicles released from lung epithelial cells. J. Cell. Physiol..

[148] Draijer C., Speth J.M., Penke L.R.K., Zaslona Z., Bazzill J.D., Lugogo N., Huang Y.J., Moon J.J., Peters-Golden M. (2020). Resident alveolar macrophage-derived vesicular SOCS3 dampens allergic airway inflammation. FASEB J..

[149] Cañas J.A., Sastre B., Mazzeo C., Fernández-Nieto M., Muñoz J.M.R., González-Guerra A., Izquierdo M., Barranco P., Quirce S., Sastre J. (2017). Exosomes from eosinophils autoregulate and promote eosinophil functions. J. Leukoc. Biol..

[150] Cañas J.A., Sastre B., Rodrigo-Muñoz J.M., Fernández-Nieto M., Barranco P., Quirce S., Sastre J., Del Pozo V. (2018). Eosinophil-derived exosomes contribute to asthma remodelling by activating structural lung cells. Clin. Exp. Allergy.

[151] Rossaint J., Kühne K., Skupski J., Van Aken H., Looney M.R., Hidalgo A., Zarbock A. (2016). Directed transport of neutrophil-derived extracellular vesicles enables platelet-mediated innate immune response. Nat. Commun..

[152] Cañas J.A., Sastre B., Muñoz J.M.R., Del Pozo V. (2019). Exosomes: A new approach to asthma pathology. Clin. Chim. Acta.

[153] Lu T.X., Munitz A., Rothenberg M.E. (2009). MicroRNA-21 Is Up-Regulated in Allergic Airway Inflammation and Regulates IL-12p35 Expression. J. Immunol..

[154] Elbehidy R.M., Youssef D.M., El-Shal A.S., Shalaby S.M., Sherbiny H.S., Sherief L.M., Akeel N.E. (2016). MicroRNA–21 as a novel biomarker in diagnosis and response to therapy in asthmatic children. Mol. Immunol..

[155] Gon Y., Maruoka S., Inoue T., Kuroda K., Yamagishi K., Kozu Y., Shikano S., Soda K., Lötvall J., Hashimoto S. (2017). Selective release of miRNAs via extracellular vesicles is associated with house-dust mite allergen-induced airway inflammation. Clin. Exp. Allergy.

[156] Moon H.-G., Kim S.-H., Gao J., Quan T., Qin Z., Osorio J.C., Rosas I.O., Wu M., Tesfaigzi Y., Jin Y. (2014). CCN1 secretion and cleavage regulate the lung epithelial cell functions after cigarette smoke. Am. J. Physiol. Lung Cell. Mol. Physiol..

[157] Shiomi T., Okada Y., Foronjy R., Schiltz J., Jaenish R., Krane S., D’Armiento J. (2003). Emphysematous Changes Are Caused by Degradation of Type III Collagen in Transgenic Mice Expressing MMP-1. Exp. Lung Res..

[158] Kasahara Y., Tuder R.M., Taraseviciene-Stewart L., Le Cras T.D., Abman S., Hirth P.K., Waltenberger J., Voelkel N.F. (2000). Inhibition of VEGF receptors causes lung cell apoptosis and emphysema. J. Clin. Investig..

[159] Li C.-J., Liu Y., Chen Y., Yu D., Williams K.J., Liu M.-L. (2013). Novel Proteolytic Microvesicles Released from Human Macrophages after Exposure to Tobacco Smoke. Am. J. Pathol..

[160] Cordazzo C., Petrini S., Neri T., Lombardi S., Carmazzi Y., Pedrinelli R., Paggiaro P., Celi A. (2014). Rapid shedding of proinflammatory microparticles by human mononuclear cells exposed to cigarette smoke is dependent on Ca2+ mobilization. Inflamm. Res..

[161] Kaur G., Singh K., Maremanda K., Li D., Chand H.S., Rahman I. (2020). Differential Plasma Exosomal Long Non-Coding RNAs Expression Profiles and Their Emerging Role in E-Cigarette Users, Cigarette, Waterpipe, and Dual Smokers. Plos One..

[162] He S., Chen D., Hu M., Zhang L., Liu C., Traini D., Grau G.E., Zeng Z., Lu J., Zhou G. (2019). Bronchial epithelial cell extracellular vesicles ameliorate epithelial-mesenchymal transition in COPD pathogenesis by alleviating M2 macrophage polarization. Nanomed. Nanotechnol. Biol. Med..

[163] Serban K.A., Rezania S., Petrusca D.N., Poirier C., Cao D., Justice M.J., Patel M., Tsvetkova I., Kamocki K., Mikosz A. (2016). Structural and functional characterization of endothelial microparticles released by cigarette smoke. Sci. Rep..

[164] Gordon C., Gudi K., Krause A., Sackrowitz R., Harvey B.-G., Strulovici-Barel Y., Mezey J.G., Crystal R.G. (2011). Circulating Endothelial Microparticles as a Measure of Early Lung Destruction in Cigarette Smokers. Am. J. Respir. Crit. Care Med..

[165] Liu G., Friggeri A., Yang Y., Milosevic J., Ding Q., Thannickal V.J., Kaminski N., Abraham E.H. (2010). miR-21 mediates fibrogenic activation of pulmonary fibroblasts and lung fibrosis. J. Exp. Med..

[166] Borges F.T., Melo S.A., Özdemir B.C., Kato N., Revuelta I., Miller C.A., Ii V.H.G., LeBleu V.S., Kalluri R. (2012). TGF-β1–Containing Exosomes from Injured Epithelial Cells Activate Fibroblasts to Initiate Tissue Regenerative Responses and Fibrosis. J. Am. Soc. Nephrol..

[167] Szul T., Bratcher P.E., Fraser K.B., Kong M., Tirouvanziam R., Ingersoll S., Sztul E., Rangarajan S., Blalock J.E., Xu X. (2016). Toll-Like Receptor 4 Engagement Mediates Prolyl Endopeptidase Release from Airway Epithelia via Exosomes. Am. J. Respir. Cell Mol. Biol..

[168] Hough K.P., Wilson L.S., Trevor J.L., Strenkowski J.G., Maina N., Kim Y.-I., Spell M.L., Wang Y., Chanda D., Dager J.R. (2018). Unique Lipid Signatures of Extracellular Vesicles from the Airways of Asthmatics. Sci. Rep..

[169] Lässer C., E O’Neil S., Ekerljung L., Ekström K., Sjöstrand M., Lötvall J. (2011). RNA-containing Exosomes in Human Nasal Secretions. Am. J. Rhinol. Allergy.

[170] Lässer C., O’Neil S.E., Shelke G.V., Sihlbom C., Hansson S.F., Gho Y.S., Lundbäck B., Lötvall J. (2016). Exosomes in the nose induce immune cell trafficking and harbour an altered protein cargo in chronic airway inflammation. J. Transl. Med..

[171] Zhang W., Zhang J., Cheng L., Ni H., You B., Shan Y., Bao L., Wu D., Zhang T., Yue H. (2018). A disintegrin and metalloprotease 10-containing exosomes derived from nasal polyps promote angiogenesis and vascular permeability. Mol. Med. Rep..

[172] Cruz F.F., Borg Z.D., Goodwin M., Sokocevic D., Wagner D.E., Coffey A., Antunes M., Robinson K.L., Mitsialis S.A., Kourembanas S. (2015). Systemic Administration of Human Bone Marrow-Derived Mesenchymal Stromal Cell Extracellular Vesicles AmelioratesAspergillusHyphal Extract-Induced Allergic Airway Inflammation in Immunocompetent Mice. Stem Cells Transl. Med..

[173] Fang S., Zhang H., Wang C., He B., Liu X., Meng X., Peng Y., Xu Z., Fan X., Wu Z. (2020). Small extracellular vesicles derived from human mesenchymal stromal cells prevent group 2 innate lymphoid cell-dominant allergic airway inflammation through delivery of miR-146a-5p. J. Extracell. Vesicles.

[174] Du Y.-M., Zhuansun Y.-X., Chen R., Lin L., Lin Y., Li J.-G., Yu-Mo D., Yong-Xun Z., Rui C., Ying L. (2018). Mesenchymal stem cell exosomes promote immunosuppression of regulatory T cells in asthma. Exp. Cell Res..

[175] Burke H., Freeman A., Ostridge K., Staples K.J., Spalluto M., Wilkinson T. (2019). Lung exosomal miRNAs discriminate between healthy ex-smokers and COPD. Airway Cell Biology and Immunopathology.

[176] Mohamed A., Pekoz A.Y., Ross K., Hutcheon G.A., Saleem I.Y. (2019). Pulmonary delivery of Nanocomposite Microparticles (NCMPs) incorporating miR-146a for treatment of COPD. Int. J. Pharm..

[177] Zhang D., Lee H., Wang X., Rai A., Groot M., Jin Y. (2018). Exosome-Mediated Small RNA Delivery: A Novel Therapeutic Approach for Inflammatory Lung Responses. Mol. Ther..

[178] Li M., Jiang M., Meng J., Tao L. (2020). Exosomes: Carriers of Pro-Fibrotic Signals and Therapeutic Targets in Fibrosis. Curr. Pharm. Des..

[179] Mansouri N., Willis G.R., Fernandez-Gonzalez A., Reis M., Nassiri S., Mitsialis S.A., Kourembanas S. (2019). Mesenchymal stromal cell exosomes prevent and revert experimental pulmonary fibrosis through modulation of monocyte phenotypes. JCI Insight.

[180] Shentu T.-P., Huang T.-S., Cernelc-Kohan M., Chan J., Wong S.S., Espinoza C.R., Tan C., Gramaglia I., Van Der Heyde H., Chien S. (2017). Thy-1 dependent uptake of mesenchymal stem cell-derived extracellular vesicles blocks myofibroblastic differentiation. Sci. Rep..

